# Histopathological diagnosis of strongyloidiasis hyperinfection in Tunisian patient with hodgkin lymphoma: Case report

**DOI:** 10.1016/j.amsu.2021.102367

**Published:** 2021-05-13

**Authors:** Ahlem Bdioui, Ahlem Bchir, Nabiha Missaoui, Sihem Hmissa, Moncef Mokni

**Affiliations:** a Pathology Department, Sahloul University Hospital, 4002, Sousse, Tunisia; b Pathology Department, Farhat Hached University Hospital, 4000, Sousse, Tunisia; c Faculty of Sciences and Techniques of Sidi Bouzid, Kairouan University, Kairouan, Tunisia

**Keywords:** *Strongyloides stercoralis*, Gastrointestinal infection, Hodgkin lymphoma, Histopathology, Diagnosis, Case report, BEACOPP, Bleomycin, etoposide, doxorubicin, cyclophosphamide, vincristine, procarbazine, and prednisone, HTLV1, Human T-lymphotropic virus, HIV, Human immunodeficiency virus, M.O.P.P, Nitrogen mustard, oncovin, prednisone, procarbazine

## Abstract

**Introduction:**

*Strongyloides stercoralis*, an intestinal nematode, is commonly dispersed throughout the tropical and subtropical regions. *Strongyloides stercoralis* infection typically contributes to an asymptomatic chronic disease which can remain hidden for decades. However, in immunocompromised patients, the hyperinfection can take place, causing high mortality rates.

**Case presentation:**

A 45 year-old Tunisian women, with heavy medical history, suffering of stage 3 classic Hodgkin lymphoma under treatment; presented with complaints of epigastric pain, nausea, vomiting. Gastroduodenoscopy showed duodenal and gastric erythematous and ulcerated mucosa. Histological assessment showed chronic infiltration with a large amount of eosinophils around numerous helminth forms identified as larvae of *Strongyloides stercoralis.*

**Conclusion:**

Early detection of *Strongyloides stercoralis* infection in immunocompromised patients is life saving and avoids fatality caused by hyperinfection or systemic dissemination. Routine stool examination may be negative, so histopathological identification of the parasite in tissue sections provides the definite diagnosis.

## Highlights

• *Strongyloides stercoralis* is very rare, prevention is necessary in the immunocompromised subject.

## 1 Introduction

*Strongyloides Stercoralis* constitutes a parasitic disease widespread in subtropical and tropical regions. In uncomplicated forms, the disease is either asymptomatic or responsible for non-specific minor signs. However, in immunocompromised patients, it is responsible for severe and fatal forms. Histopathological detection of the parasite in tissue section seems to be the best way to provide the definite diagnosis [1,2]. Below, we report a case of a patient suffering from Hodgkin lymphoma diagnosed with strongyloidiasis hyperinfection. We had followed the instruction of 2020 scare guidelines [3].

## 2 Case presentation

We presented a 45-year-old Tunisian woman, with a heavy past medical history of orbital cellulitis, peptic esophagitis, two years of unexplained prolonged fever with chronic pruritus, and suffering from grade 2 rheumatic mitral regurgitation. There was no family history and no drugs allergic or psychosocial history.

The subject was also operated for uterine leiomyoma and acute appendicitis, seven and three months respectively before the current episode. She was from a rural area, never travelled abroad and didn't report history of farm work.

The patient was diagnosed two months earlier with stage III classic Hodgkin's lymphoma. She received a first BEACOPP (bleomycin, etoposide, doxorubicin, cyclophosphamide, vincristine, procarbazine, and prednisone) escalated chemotherapy cycle and the second cycle of chemotherapy was postponed since the patient presented a fistulised cervical adenopathy. A few days later, she experienced epigastric pain, vomiting with profuse diarrhea. After consulting the emergency, she was referred to the Infectious Diseases Unit for suspicion of infectious colitis. The patient was then transferred to the Intensive Care Unit, since she developed a hypovolemic shock. A digestive lymphomatous infiltration was suspected. Thus, an oesophago-gastro-duodenoscopy was performed and showed duodenal and gastric erythematous and ulcerated mucosa.

**Fig. 1:**
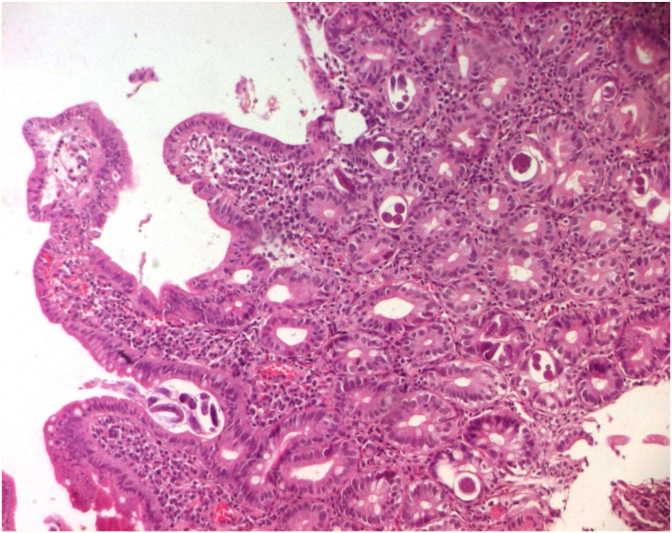
Duodenal mucosa showing cross-section of *Strongyloides stercoralis* (HE x 100).

**Fig. 2:**
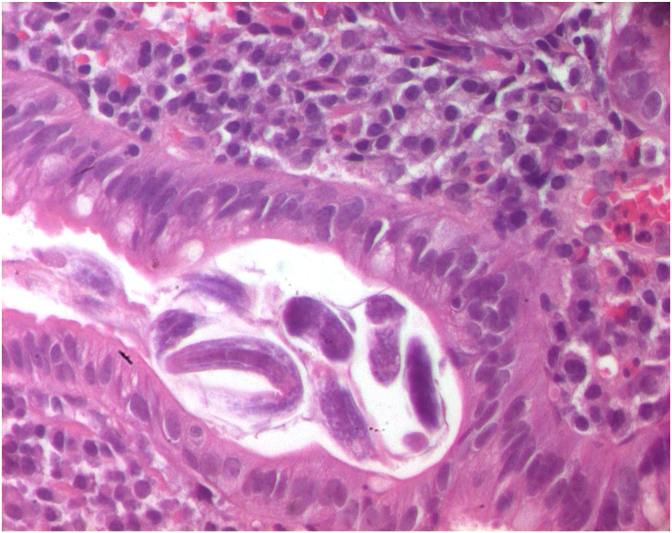
Cross-section of *Strongyloides stercoralis* (HE x 400).

**Fig. 3:**
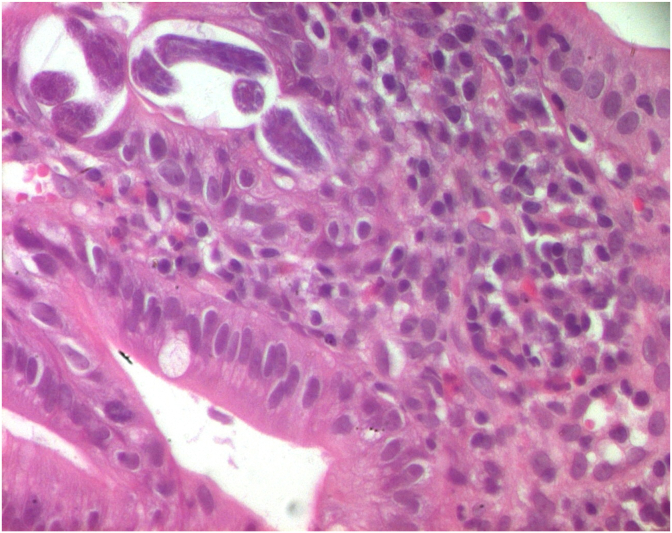
Chronic infiltration with a large amount of eosinophils (arrow) around numerous helminthes forms (HE x 400).

Histological assessment showed numerous helminth forms identified as larvae of *Strongyloides stercoralis* (Fig. 1, Fig. 2), surrounded by chronic infiltration with a large amount of eosinophils (Fig. 3).

The diagnosis of strongyloidiasis was confirmed. Albendazole was administered to the patient. In spite of anti-parasite treatment, the patient's condition was worsening. Two weeks after her admission she died with multi-organ failure.

## 3 Discussion

*Strongyloides stercoralis* is a parasitic disease widespread in tropical and subtropical countries. One of the particularities of our case is its occurrence in a Tunisian woman. However, Tunisia is neither tropical nor known to be endemic for this parasite.

The parasitic cycle is complex. Humans are affected by contacting contaminated soil. The filariform larvae infiltrate the host skin, travel to the lungs by way of the blood circulation and then penetrate the alveolar spaces. Subsequently, they are transferred to the pharynx, eventually ingested, and attain the small intestine. The larvae molt twice to become adult female worms that generate eggs in the intestinal mucosa. Once they hatch, the new rhabditiform larvae travel to the lumen. From there, they reach the stool or cause autoinfection [2].

In uncomplicated forms, the disease is either asymptomatic or responsible for non-specific minor signs. Pruritus is usually the first symptom of the disease. This was observed in our case since the patient had two year history of pruritus. Then, it is associated with respiratory symptoms, in particular coughing and shortness of breath. Digestive signs appear when the parasite reaches the intestine. They include diffuse abdominal pain, nausea and vomiting. Paralytic ileus and exudative entheropathy have also been described [1].

Noticing these non-specific signs, *Strongyloides stercoralis is* a very elusive and confusing disease. Most cases reported in non-endemic area, particularly didn't suspect the infection, as described in our case, and the parasite infestation was discovered incidentally.

In immunocompromised patients, the infection is responsible for serious or even fatal forms as the parasite didn't follow the usual route (skin, lungs, intestine); but it is diffused throughout the whole body and affects several organs. This is known as hyperinfection, occurring in only 2.5% of cases. These serious forms associate to the digestive and respiratory signs a multi-visceral failure with fever, deterioration of the general condition, respiratory distress, renal insufficiency and neurological disorder [1]. Concerning our patient, hyperinfection symptoms included abundant diarrhea, general condition deterioration and hypovolemic shock.

Several causes of immunodepression associated with hyperinfection have been reported such as diabetes, chronic obstructive pulmonary syndrome, alcoholism, chronic renal failure, hypogammaglobulinemia, malnutrition, and malignancy. However, the most frequent condition is the use of immunosuppressive therapy, particularly glucocorticoids, known to suppress eosinophils and inactivate lymphocytes. Methotrexate and Ritoximab were also involved [1,2,4,5]. Co-infection with the human T-lymphotropic virus (HTLV1) virus has been also cited; whereas, involvement of the human immunodeficiency virus (HIV) infection is controversial [[6], [7], [8]].

Similar to our case who was associated with Hodgkin lymphoma under treatment, other cases have been diagnosed in association with hematologic malignancy [[9], [10], [11]]. Adam et al. [10] reported three cases of hyperinfection associated with lymphoma (one of them was classic Hodgkin lymphoma) treated with M.O.P.P. (nitrogen mustard, oncovin, prednisone, procarbazine) combination therapy. But, the strongyloides hyperinfection was not diagnosed until necropsy [10]. Yacin et al. [11] report a case of disseminated strongyloidiasis following steroids and radiotherapy treatment of myeloma. The patient developed hyperinfection with fatal outcome, even the Ivermectin and Albendazole treatment [11].

Most authors link hyperinfection in hematologic malignancy to the use of glucosteroids [1,2,[4], [5], [6], [7], [8], [9], [10], [11]]. However, Incani [2] suggested a relationship between hyperinfection and Rituximab. In fact, he report a case of patient with Mantle-cell lymphoma, who developed hyperinfection long time after the ending of the chemotherapy cycle, and the patient has received then only Retixumab [2].

On the other hand, in two of the three patients having proved lymphoma reported by Adam et al. [10], hyperinfection has occurred before lymphoma drug therapy. Thus, a partial immunological paresis, that is known to occur in lymphoma, could explain the hyperinfection [10].

Diagnosis confirmation is based on the revelation of the parasite. Stool examination is the first line exam. However, it has a low sensitivity since the intestinal larva consignment is low and the larval output is minimal. Thus, it must be remade several times in order to isolate the parasite [1]. Microscopic examination of duodenal biopsy or bronchoalveolar wash enables to demonstrate the parasites and evaluate the extent of the inflammatory response [2]. Enzyme-linked immunosorbent assay (ELISA) is another alternative for diagnosis. However, it is expensive as well as it does not discriminate between old and recent forms and it can show a cross-reaction with other helminths [2]. Indeed, histopathological exam seems to be the more appropriate tool to provide the final diagnosis.

Uncomplicated forms can be treated by Thiabendazole, Ivermectin and Albendazole; repetition of the first course is highly recommended ensuring eradication. For hyperinfection, Ivermectin in daily use until parasite eradication constitute the alternative treatment [12,13]. In order to confirm parasite suppression, the follow-up stool examination should be carried out over a period of three months after the management [11].

## 4 Conclusion

*Strongyloides stercoralis* is an innocuous parasitic disease. However, in immunocompromised subjects, it is responsible for serious and fatal form. Thus, identification of the parasite before starting immunosupressor treatment is highly recommended, especially in endemic areas.

## Please state any conflicts of interest

No conflicts of interest

## Please state any sources of funding for your research

This research did not receive any specific grant from funding agencies in the public, commercial, or not-for-profit sectors.

## Sources of funding

This research did not receive any specific grant from funding agencies in the public, commercial, or not-for-profit sectors.

## Ethical approval

This study is exempt from ethical approval at our institution.

## Consent

Written informed consent was obtained from the patient for publication of this case report and accompanying images. A copy of the written consent is available for review by the Editor-in-Chief of this journal on request.

## Author contribution

Ahlem Bdioui Thabet: analyzed and interpreted the patient data, she was responsible for the conception and design. Nabiha Missaoui and Ahlem Bchir: participate to the acquisition and interpretation of data. Sihem Hmissa and Moncef Mokni: have drafted the work and revised it. All authors read and approved the final manuscript.

## Trial registry number

1. Name of the registry:
2. Unique Identifying number or registration ID:
3. Hyperlink to your specific registration (must be publicly accessible and will be checked):

## Guarantor

Ahlem Bdioui.

## Declaration of competing interest

No conflicts of interest.

## References

[1] Altintop L., Cakar B., Hokelek M., Bektas A., Yildiz L., Karaoglanoglu M. (2010). Strongyloides stercoralis hyperinfection in a patient with rheumatoid arthritis and bronchialasthma: a case report. Ann. Clin. Microbiol. Antimicrob..

[2] Al-Sajee D., Al-Hamdani A. (2010). A case of gastric and duodenal strongyloidiasis. Sultan Qaboos Univ Med J.

[3] Agha R.A., Franchi T., Sohrabi C., Mathew G., pour le groupe S.C.A.R.E. (2020). The SCARE 2020 guideline: updating consensus surgical CAse REport (SCARE) guidelines. Int. J. Surg..

[4] Rothe K., Katchanov J., Schneider J., Spinner C.D., Phillip V., Busch D.H. (2020). Strongyloides stercoralis hyperinfection syndrome presenting as mechanical ileus after short-course oral steroids for chronic obstructive pulmonary disease (COPD) exacerbation. Parasitol. Int..

[5] GeethaBanu S., Arthi K. (2020). Strongyloides hyperinfection in a patient with solid malignant tumor a case report. Indian J. Publ. Health.

[6] Martyn E., Gration B., Somasundaram C., Chiodini P.L. (2019). Strongyloides, HTLV-1 and small bowel obstruction. BMJ Case Rep..

[7] Alpern J.D., Arbefeville S.S., Vercellotti G., Ferrieri P. (2017). Green JS Strongyloides hyperinfection following hematopoietic stem cell transplant in a patient with HTLV-1-associated T-cell leukemia. Transpl. Infect. Dis..

[8] Siegel M., Simon G. (2012). Is human immunodeficiency virus infection a risk factor for strongyloides stercoralis hyperinfection and dissemination. PLoS Neglected Trop. Dis..

[9] Incani R.N., Hernández M., González M.E. (2010). Hyperinfection by *Strongyloides stercoralis* probably associated with rituximab in a patient with mantle cell lymphoma and hyper eosinophilia. Rev. Inst. Med. Trop. Sao Paulo.

[10] Adam M., Morgan O., Persaud C., Gibbs W.N. (1973). Hyperinfection syndrome with Strongyloides stercoralis in malignant lymphoma. Br. Med. J..

[11] Yassin M.A., El Omri H., Al-Hijji I., Taha R., Hassan R., Al Aboudi K., El-Ayoubi H. (2010). Fatal *Strongyloides stercoralis* hyper-infection in a patient with multiple myeloma. Braz. J. Infect. Dis..

[12] Pacanowski J., Santos M.D., Roux A., Le Maignan C., Guillot J., Lavarde V., Cornet M. (2005). Subcutaneous ivermectin as a safe salvage therapy in Strongyloides stercoralis hyperinfection syndrome: a case report. Am. J. Trop. Med. Hyg..

[13] Grossi P.A., Lombardi D., Petrolo A., Rovelli C., Di Rosa Z., Perriccioli G., Rossi A., Minoja G., Scaglione F., Dalla Gasperina D. (2018). Strongyloides stercoralis hyperinfection in an HIV-infected patient successfully treated with subcutaneous ivermectin. Trav. Med. Infect. Dis..

